# Laparoscopic treatment of a Yolk Sac Tumor: case report and literature review

**Published:** 2013-09-02

**Authors:** Maurizio Guida, Sandro Pignata, Anna Rita Palumbo, Gianmarco Miele, Maria Luisa Marra, Federica Visconti, Fulvio Zullo

**Affiliations:** 1 Department of Gynaecology and Obstetrics, University of Salerno, Salerno, Italy.; 2 Department of urology and gynecology, National Cancer Institute, Naples, Italy

**Keywords:** ovary, yolk sac tumor, laparoscopic surgery, fertility

## Abstract

We report the case of a yolk sac tumor of the ovary in a 26 years old woman. The laparoscopic approach and the BEP chemotherapy were fundamental to have a minimally invasive treatment and to preserve fertility.

## INTRODUCION

The ovarian germ cell tumors (OGCTs) [ tab.1 ] represent 15–20% of all ovarian tumors [ 1 ] and originate from the primitive germ cell and then gradually differentiate to mimic the developmental tissues of embryonic origin (ectoderm, mesoderm, endoderm), and the extraembryonic tissues (yolk sac and trophoblast). Malignant Ovarian germ cell tumors (MOGCTs) constitute 3–5% of all ovarian malignancies [ 2 , 3 ] . OGCTs are subdivided into germinomatous e non-germinomatous tumors. The most common types of non-germinomatous tumors are yolk sac and immature teratoma. As regard as ovarian yolk sac tumors (YST) altough rare, are the second most frequent histological subtype of MOGCTs, after ovarian dysgerminoma. They account for 20% of MOGCTs [ 4 ] and are frequent especially in childhood and in early adulthood, between 18–30 years old woman [ 5 ] . For this reason the treatment of yolk sac tumors is finalized to preserve the fertility and therefore it is imperative that these tumors are managed with accurate diagnosis, staging and treatment.

We report here the case of a 26 years old women with a YST treated with laparoscopic technique with success.

## CASE-REPORT

A 26 years old female with body height of 1,68 m was admitted to our department with abdominal pain. She referred us a history of intermittent abdominal pain which had slowly been worsening over three month. In the last month there was loss of appetite. She reported amenorrhea for the past two cycles. She had no important past medical or surgical history. On palpation a vague mass was felt in the lower abdomen. The vaginal examination revealed a mass in the left fornix that was mobile and sore.

After the visit we executed an ultrasound examination with 3.5 MHz curvilinear probe that revealed a large mix echogenic mass measuring approximately 4cm in mayor diameter and 2,5 cm in smaller arising from pelvis that appears multilocular, cystic and solid in nature. At color doppler examination there was increased vascularity noted in the solid component of mass. No evidence of calcification. No fluid in the Douglas. The origin of the mass could not be ascertained, but was felt to be likely from the left ovary because a separate, normal left ovary could not be visualized. The right ovary appeared normally with the presence of a follicle of 1,5 cm.

Biochemical and laboratory investigations were made. Labs included a hemoglobin of 12,1 g/dl, a hematocrit of 37,9% and platelets of 275.000; creatinine was normal (0,76 mg/dl) and also BUN (32 mg/dl); liver function tests that included bilirubin (0,54 mg/dl), aspartate aminotrasferase (19 u/L), alanine aminotrasferase (17 u/L) were normal; Serum human gonadotropin hormone levels (4,2 mUI/ml) and CA-125 levels (293 UI/L) were within normal limits. A markedly elevated serum alpha-fetoprotein (AFP) level to 18.178 ng/ml oriented toward the diagnosis of an ovarian yolk sac tumor.

Subsequently, CT of the abdomen was performed. A 4,5 cm size complex mass lesion in the left ovary with solid component and cystic areas adjacent to it was present. Pelvic and paraahortic lymph nodes were in the range limits for the dimension and morphology. No calcified peritoneal implants were apparent, the others abdomen bowels appeared of normal structure.

After our evaluations we decided to plan a laparoscopic surgery. We provide the steps of surgery; after the creation of pneumoperitoneum with Verres needle, a central trocar was used for the optics, others three trocars were inserted as lateral operative accesses. The uterus and right adnexa appeared normal with no lesion; the left adnexa showed a large mass with cystic and necrotic aspects. There was no peritoneal carcinomatosis and no lesions on the liver or subdiaphragmatic areas. A peritoneal washing was made; After a total omentectomy, obturator and paraahortic lymph nodes dissection were also added to the left salpingo-oophorectomy; a biopsy of right ovary, of left parieto-colic sulcus and appendicectomy were made. Specimens were sent for histopathological examination [ Tab2 ]. The left ovary mass showed tumor cells arranged in papillary groups with a glandular pattern and acinar structure. The lining of the papillary infolding was irregular. Also there was the presence of Schiller-Duvall body, typical patterns of YST. The postoperative course was regular and the alphafetoprotein levels decreased rapidly. It was planned a chemotherapy with the BEP regimen (Bleomycin, Etoposide, Cisplatin). A total of two cycles of chemotherapy were repeated every 3 weeks. Serum AFP level had fallen to normal range 4 weeks after the surgery.

## DISCUSSION

Yolk sac tumor is also called endodermal sinus tumor (EST) because there is a link to its discovery. Histologically the malignant tissue resemble structure found in early embryonic development, and it is Shiller’s stressing of the unique glomeruloid structure that led Telium to compare it with the yolk sac endodermal sinuses of Duval in the rat placenta [ 6 ] .

High values of AFP orientate strongly to diagnosis of YST. Furthermore it is a sensible marker for tumor’s evolution; infact a rapid decrease of AFP levels in the serum after surgery is a sign of absence of residual tumor. Also the efficiency of chemotherapy its related with normalization of AFP [ 8 ] . It is almost always unilateral and large with a diameter that may vary from 5 to 50 cm (median 15 to 19 cm) [ 9 ] . The typical neoplasm manifest as a large complex pelvic mass that extends into the abdomen. This tumor is often characterized by extremely rapid growth and extensive intra-abdominal spreading with poor prognosis [ 10 ] .

As regard clinical, abdominal pain is the principal symptom leading the discovery of the disease which may require the emergency surgery especially in cases of ovarian torsion [ 11 ] . Other symptoms are the presence of an abdominal or pelvic mass with abdominal enlargement, vaginal bleeding, fever, ascites or peritonitis secondary to torsion, infection or rupture of the ovarian tumor [ 3 , 8 ] . In consequence of ascites it’s possible to have decrease breath sounds in the bilateral lung bases, diffuse tenderness to palpation of the abdomen and distant bowel sound [ 12 ] .

Preoperative radiological diagnosis is difficult. Yolk sac tumors can be cystic, with signs of hypervascularization and areas of haemorrhage. However YST haven’t a specific image both ultrasound that MRI and CT. If YST appear with a solid portion, multiple small arterioles with lower RI are detectable by color Doppler ultrasound, haemorrhagic spots can be demonstrated by T1 weighted MRI, hypervascularity can be shown on contrast-enhanced T1 weighted scans [ 7 ] . The malignant evolution of YST consist in locoregional extension involving uterus, pelvic peritoneum, rectum and bladder. Other authors described the involvement of the omentum, abdominal peritoneum and serosal surfaces of bowel in 30% of the cases. Retroperitoneal lymph nodes and liver parenchyma were also involved in advanced stages. [ 3 , 13 , 14 ]

The standard management of malignant ovarian germ cell tumors is complete surgical excision.

Because of most of them are unilateral, it’s possible executing a fertility sparing surgical treatment. The type of surgical procedure was not an important prognostic factor for patients with malignant germ cell tumors of the ovary at all clinical stages, and so the conservative surgery is possible if it’s followed by chemotherapy [ 15 ] .

In the past after the laparoscopic diagnosis of an ovarian cancer, laparo-conversion was recommended to have an optimal staging and to avoid an uncertain tumor cell spread. Today, advancement in technologies offers a minimally invasive surgery that recognizes in the laparoscopic surgery a new approach for ovarian cancer. It gave us not only a same or even better surgical prognosis as compared the conventional laparotomy [ 16 ] but benefit from a less traumatic surgery and a potentially faster recovery [ 17 ] .

The aim of surgery is removing the primary ovarian tumor without excessive morbidity. Thus cycles of chemotherapy should be administer while monitoring the rate of decline of serum AFP. Specifically, before the introduction of effective combination chemotherapy, the prognosis for patients with malignant nondysgerminomatous cancers was poor; as regards patients with YST, they had a 3-year survival rate of 13% [ 18 ] . Currently, initial surgery followed by adjuvant chemotherapy including bleomycin, etoposide and cisplatin (BEP) is considered the standard for the treatment of YolkSacTumor [ 19 ] .

Compared with other regimens, BEP appears to be the best active first-line option for primary, metastatic, or recurrent disease. As regards the chemotherapy-related toxicity, it was acceptable and severe collateral effects was infrequent, although myelosuppression can be observed in the majority of patients. The administration of G-CSF would help manage this toxicity. Infrequent severe pulmonary toxicity, ototoxicity, or nephrotoxicity are observed [ 20 ] .

Administration of bleomycin as a slow infusion (over 24 h)may reduce the incidence of toxicity, but this has yet to be confirmed [ 19 ] . Administration of etoposide for three days instead of five, is an effort to decrease the incidence of neutropenia without sacrificing efficacy. Etoposide has been reported to be associated with the development of an acute leukemia with typical morphological and cytogenetic features [ 22 ] . Long-term toxicity was limited and consisted in hypertension, as already described in testicular cancer survivors [ 21 – 23 ] .

Progressive or recurrent ovarian YST after treatment with BEP chemotherapy is associated with a poor prognosis. There are not approved schemes of chemotherapy in this cases, so it is possible adapt chemotherapy used for recidivist testicular cancer. Possible options of chemotherapy include combination of vinblastine, ifosfamide, cisplatinum, or paclitaxel, ifosfamide, cisplatinum, [ 24 ] . It should also be stressed that secondary cytoreductive surgery could play an important role when tumors are limited and resistant to chemotherapy.

As regards the follow-up of chemotherapy, the determination of initially elevated markers (AFP) should be repeated before each cycle of therapy, soon after the end of the treatment and during the 2 years after the end of chemotherapy [ 20 ] . An annual pelvic ultrasound is necessary in the case of conservative treatment, to screen for a contralateral recurrence [ 25 ] .

The BEP regimen, no ascites at presentation, stage I disease, less of 42 days to AFP normalization, fertilitysparing surgery and a serum AFP half-life less of 10 days are factors associated with a good prognosis.

Lumbaraortic lymphadenectomy has been proposed to identify patients with a higher risk of relapse after surgery, in order to guide the choice of adjuvant chemotherapy [ 25 ] .

In the study of de La Motte, Rouge and collaborators [ 4 ] lymphnode metastasis was found exclusively in 2 patients with stage IIIC peritoneal disease in whom chemotherapy is mandatory. No lymph node involvement was found in stage I disease.

Another important consideration is the impact of chemotherapy on gonadal and reproductive function. In the past the majority of patients with MOGCT receiving multidrug combination chemotherapy, showed a decreased number of primordial follicles and an increase in stromal fibrosis and in atrophied cortices [ 26 ] . Today for early stage disease the aim of chemotherapy is to be efficacy and at the same time to minimize toxicity and retain reproductive function [ 27 , 28] . Different studies [29, 30] reported the resume of normal menstrual function and in some patients pregnancies after BEP chemotherapy. For example, Brewer et al reported on 26 patients treated with BEP for MOGCT, 71% of which resumed normal menstrual function, and six of whom conceived [29] .

Therefore the risk of of infertility following treatment of MOGCT is always a concern, even if the majority of patients, especially in the early stage, will maintain their ovarian function and fertility. Following this strategy, most patients will be cured and be able to give birth.

## Figures and Tables

**Table 1:** Subtypes and characteristics of ovarian germ cell tumours (data derived from Rice, 1999 and John Hopkins Pathology, 2001).

Subtype	Frequency of OGCT	Benign /malignant	Uni- or bi-laterale	Tumour markers expressed	Metastasis route
Dysgerminoma	35–50 %	malignant	10–15 % are bilateral	Serum lactic dehydrogenase and serum hGC	Lymphatic system
Yolk sac tumor (YST)	20%	malignant	Usually unilateral	AFP(commonly), alpha 1 antitrypsin (rarely)	Intraperitoneally and hematogenously
Embryonal carcinoma	Rare	malignant		AFP and hGC	intraperitoneally
Polyembrioma	Rare			AFP and hGC	
Choriocarcinoma	Very rare	malignant	Usually unilateral	Hgc	
Teratoma	Immature account for 20% of MOGCTs	Benign or malignant	12–15 % are bilateral	Immature teratomas sometimes secrete AFP, serum LDH and Ca125	
Mixed GCT	10–15%	Dependent upon the cell types present		Dependent upon the cell types present	

**Table 2:** pathologist report, Department of Pathology; University of Salerno, Salerno, Italy.

Macroscopic description	Microscopic description
Left ovary with mass (4,5 cm * 2 cm) + left fallopian tube (6 cm)	Residual ovarian parenchyma and cystic spaces lined by cells with clear cytoplasm and hyperchromatic nuclei arranged in papillary groups compatible with yolk sac tumor
Appendix (3 cm)	Absence of disease
Omentum with free areas of thickening (12 cm * 4 cm) + left parieto-colic sulcus (1,5 cm)	Absence of disease
Right ovary biopsy	Ovarian parenchyma with ovarian follicles and a corpus luteum
6 paraahortic lymph nodes	Reactive hyperplasia
9 obturator lymph nodes	Reactive hyperplasia
Peritoneal fluid	Inflammatory and mesothelial cells, absence of malignant cells.
